# Gallium Resistance in *Staphylococcus aureus*: Polymorphisms and Morphology Impacting Growth in Metals, Antibiotics and Polyfluorinated Compounds

**DOI:** 10.3390/applmicrobiol5010032

**Published:** 2025-03-20

**Authors:** Akamu Ewunkem, Felicia Simpson, David Holland, Tatyana Bowers, Ariyon Bailey, Ja’nyah Gore, Uchenna Iloghalu, Vera Williams, Sarah Adjei-Fremah, Larisa Kiki, Brittany Justice

**Affiliations:** 1 Department of Biological Sciences, Winston Salem State University, Winston-Salem, NC 27110, USA; 2 Department of Mathematics, Winston Salem State University, Winston-Salem, NC 27110, USA; 3 UNC Health Care Hillsborough, Hillsborough, NC 27278, USA; 4 Department of Applied Sciences, North Carolina A and T State University, Greensboro, NC 27411, USA

**Keywords:** gallium, polymorphisms, selection, *Staphylococcus aureus*, metals, polyfluorinated compounds

## Abstract

**Background and Objectives:**

The imminent threat of antibiotic resistance has spurred studies of nonconventional antimicrobial approaches. Gallium utilization is a promising and emerging approach to treating a variety of resistant bacteria using “Trojan horse” strategies to disrupt iron-dependent processes and biofilms. This study utilized experimental evolution to test the evolvability of gallium resistance in *Staphylococcus aureus* and resistance traits potentially correlated with metals, antibiotics and polyfluorinated compounds, as well as its genomics foundations.

**Methods:**

Whole-genome sequencing was utilized to reveal functional networks of mutations associated with gallium resistance. Additionally, scanning electron microscopy (SEM) observation was utilized to visualize distinct morphological changes on the surface of gallium-resistant populations and compare with the control populations.

**Results:**

As demonstrated by these studies, *S. aureus* evolved resistance to gallium after 20 days of selection. Furthermore, these populations displayed resistance traits correlated with heavy metals and polyfluorinated compounds. In contrast, the gallium-resistant populations were very sensitive to antibiotics. Whole-genome analysis revealed significant polymorphisms in the gallium (III)-resistant populations for example, polymorphisms in staphyloferrinA export MFS transporter/D ornithine citrate ligase (*sfaA/sfaD*), teichoic acid D Ala esterase (*fmtA*), DUF3169 family protein (*KQ76_RS01520*) and adenine phosphoribosyltransferase (*KQ76_RS08360*), while polymorphisms in the ABC transporter permease subunit (*pstC*) and acyltransferase family protein (*KQ76_RS04365*) were unique to the control populations. The polymorphisms directly affected the cells’ morphology. SEM images showed significant external ultrastructural changes in the gallium-selected bacterial cells compared to the control cells.

**Conclusions:**

Our study confirmed that using gallium as an antimicrobial can have significant health and environmental implications.

## 1. Introduction

Bacterial infections are increasingly harder and difficult to treat with antibiotics and remain a significant challenge in clinical medicine. Antibiotics provide a protective umbrella to treat bacterial infections [1]. However, bacteria can acquire resistance to one or more antibiotics [2]. Consequently, new approaches in the development of the next generation of antibiotics are exigently needed. Recently, much attention has been focused on the need for new antimicrobial agents such as heavy metals [3].

Most heavy metals are naturally occurring elements and possess antibacterial activities due to their ability to inactivate proteins and enzymes through inappropriate binding of metal-binding sites in enzymes [4,5]. Heavy metals catalyze and activate the production of reactive oxygen species (ROS), leading to oxidative stress in bacterial cells, damaging nucleic acids, proteins and lipids, which are absolutely crucial for bacterial survival [3,6]. Furthermore, heavy metals can interfere with bacterial cell walls, making cells more permeable to often-toxic oxygen compounds [7]. As bacterial resistance to existing antibiotics is on the rise, which is leading to an increase in morbidity and mortality, the discovery of metals as new alternative antimicrobial agents is paramount now more than ever. Gallium has garnered interest for its ability to inhibit bacterial growth by disrupting bacterial iron metabolism [8–10].

Gallium is a promising antimicrobial agent with excellent antibacterial effects, including activity against multidrug-resistant bacteria by interrupting iron homeostasis [11,12]. Gallium is a group IIIA metal with atomic number 31 and shows exceptional hallmarks compared to other metals. Gallium exhibits good biodegradability and shape deformability, low toxicity, and high electrical and thermal conductivity suitable for in vivo applications [13]. Other than its antimicrobial activity, gallium is as an excellent drug carrier for tumor treatment [14].

Gallium has similar properties to iron in that it has an atomic radius of 62 pm, an octahedral ionic radius of 0.620 Å and a tetrahedral ionic radius of 0.47 Å; due to these similarities, gallium can bond with iron-binding proteins [15]. This property allows gallium to gain access to bacterial cells interfering with iron-dependent enzymes, rendering gallium a promising “Trojan horse” [16]. Targeting bacterial iron metabolism is superior to conventional antibiotics in alleviating the development of drug resistance [17].

Gallium has shown promising antimicrobial activity against *S. aureus* by targeting multiple heme/iron-dependent metabolic pathways, cell walls and membrane proteins [18,19]. *S. aureus* resistance to gallium has never been recorded. In contrast, there is evidence of gallium resistance in Gram-negative bacteria. Graves et al. [20] reported that *E. coli* rapidly can develop resistance to gallium via acquisition of mutations. Thus, like other antimicrobials, at some point, *S. aureus* may develop resistance to gallium, making gallium ineffective in treating *S. aureus* infections. Alternatively, bacteria resistant to gallium may be considered relevant in terms of biodegradation of polyfluorinated compounds. Polyfluorinated compounds, also known as “forever chemicals”, are an environmentally daunting class of synthetic chemicals which, under typical environmental conditions, are not broken down, resulting in costly environmental remediation efforts [21]. The presence of polyfluorinated compounds increases the risk of cancers and health issues [22]. Scientists are endlessly researching specific microbial species that can biodegrade polyfluorinated compounds into less toxic forms [23]. Yet no studies have considered the possibility of *S. aureus* evolving resistance to gallium or the ability of *S. aureus* to acquire correlated resistance to heavy metals, antibiotics and polyfluorinated compounds.

Therefore, in this study we used *Staphylococcus aureus* to deduce how Gram-positive bacteria evolve resistance to gallium and evaluated the genomic and morphological changes of this adaptation. Furthermore, we investigated whether *S. aureus* adaptation resulted in resistance or susceptibility to increasing concentrations of metals, antibiotics and polyfluorinated compounds. To the best of our knowledge, gallium resistance in *S. aureus* and correlated resistance to metals, antibiotics and polyfluorinated compounds have never been studied, thus highlighting the novelty of this study.

## 2. Materials and Methods

### 2.1. Materials

Gallium (III) nitrate (60–1000 mg/L) (99.999% anhydrous; catalog number 032116.14; Thermo Scientific^™^, Hampton, NH, USA), iron (III) nitrate hydrate (60–1000 mg/L) (Puratronic^™^, 99.999%; catalog number 197810020; Thermo Scientific, Hampton, NH, USA), 96-well plates (catalog number 269787; Thermo Scientific^™^, Hampton, NH, USA), silver chloride (6.0–50.0 mg/L) (99.9%; catalog number 011421.14; Thermo Scientific, Hampton, NH, USA), 50 mL Erlenmeyer flasks (catalog number 4117-0125; Thermo Scientific^™^, Hampton, NH, USA), nutrient broth (Thermo Scientific^™^, Hampton, NH, USA), the GloMax^®^-Multi Microplate (catalog number TM297, Promega, Madison, WI, USA) and polyfluorinated compounds (60–1000 mg/L) (GenX, a synthetic chemical compound, and perfluorooctanesulfonic acid (PFOS)) were a gift from a collaborator. Chloramphenicol (6.0–125.0 mg/L) (98%; catalog number B20841.14; Thermo Fisher Scientific, Hampton, NH, USA) and tetracycline concentrations of 6.0–125.0 mg/L (catalog number AAJ6171406; Fisher Scientific, Hampton, NH, USA) were also used.

### 2.2. Bacterial Strains and Growth Conditions

*Staphylococcus aureus* (ATCC 25923) (NCBI gene ID: https://www.ncbi.nlm.nih.gov/nuccore/CP009361.1?report=fasta; accessed on 3 March 2025) strains were cultured in nutrient broth media in 50 mL Erlenmeyer flasks at 37 °C for 24 h with shaking (150 RPM) for experimental purposes.

### 2.3. Minimum Inhibitory Concentration (MIC) of Gallium Nitrate

The minimum inhibitory concentration (MIC) of gallium nitrate was determined by serial dilution in nutrient broth (NB). An overnight culture was diluted with 0.05 O. D_650nm_ and transferred to 96-well plates in triplicate at concentrations ranging from 0 to 500 mg/L of gallium (III) nitrate. Bacterial growth in NB was assessed by measuring turbidity at O. D_650nm_ nm for 0 and 24 h in the GloMax^®^-Multi Microplate using clear polyester 98-well plates. The O. D_650nm_ measurement readings were mathematically subtracted from the values recorded at 24 h and for statistical analysis.

### 2.4. Experimental Evolution

**Figure 1.**
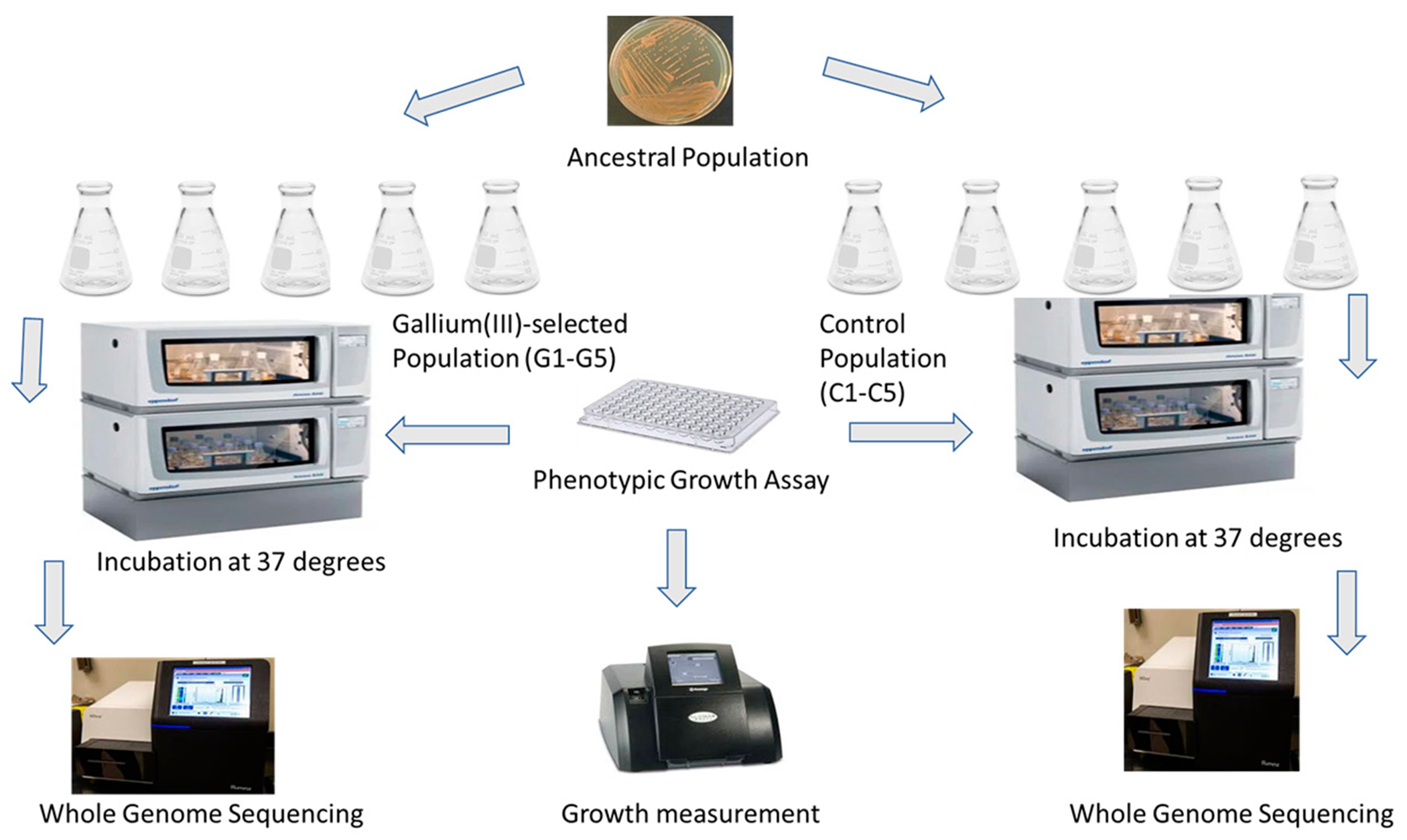
Experimental evolution layout.

The stock culture was propagated daily by transferring 0.1 mL of each culture into 9.9 mL of fresh NB for 7 days of regrowth prior to selection for gallium (III) nitrate. The controls were set up by transferring five different 0.1 mL samples and adding them to 9.9 mL of NB. The bacterial cultures were grown for 24 h, representing approximately 48 generations, since *S. aureus* is known for its quick division rate, with the ability to replicate every 30 min in ideal laboratory settings [24]. Experimental evolution by the serial transfer method was conducted in this study according to the broth microdilution method [20,25]. *Staphylococcus aureus* (ATCC 25923) colonies were plated from a stock solution onto NB agar, and individual colonies were isolated via serial dilution. The individual colonies were used to found 10 bacterial strains in sterile NB in 50 mL Erlenmeyer flasks. Five flasks (C1–C5) were designated as the control group, receiving only NB and the *S. aureus* strain, while 5 flasks (G1–G5) were designated as the treatment group and were exposed to gallium (III) over time (Figure 1). A serial transfer protocol was used, allowing cultures to grow for 24 h before transfer to new sterile media. Control group flasks received 9.9 mL of NB and 0.1 mL of the previous culture every 24 h. The treatment group was propagated using the same protocol, in addition to an increasing amount of gallium nitrate (300 mg/L) over the course of the study. The inoculated flasks were kept daily inside a 37 °C shaking incubator at 150 RPM.

### 2.5. Growth Assays: 24 h Growth

At 20 days of evolution, the gallium (III)-selected populations, along with the control populations, were assessed for fitness and adaptability in increasing concentrations of gallium (III) nitrate, iron (III) nitrate, Genx, perfluorooctanesulfonic acid (PFOS), chloramphenicol and tetracycline. These values were compared with the five samples of the *S. aureus* ancestor, which grew overnight in NB. The range used was 0–1000 mg/L for gallium (III), 0–1750 mg/L for iron (III), 0–100 mg/L for silver, and finally 0–500 nM for Genx, a synthetic chemical compound, and perfluorooctanesulfonic acid (PFOS). Bacterial growth in NB was assessed by measuring turbidity at O. D_650nm_ nm for 0 and 24 h in a GloMax^®^-Multi Microplate using clear polyester 98-well plates (Figure 1). The O. D_650nm_ measurement readings at 0 h were subtracted from the 24 h readings for statistical analysis.

### 2.6. Scanning Electron Microscopy (SEM) Sample Processing

Morphological characteristics of the gallium (III)-resistant bacterial, control and ancestral cells were imaged at 20 days of selection under a Carl Zeiss Auriga-BU FIB FESEM (FESEM) (Carl Zeiss, Jona, Germany) [25–30]. Briefly, the bacterial suspensions were centrifuged, and the precipitated bacteria were suspended in PBS (0.1 M, pH 7.2). The samples were then fixed within 2.5% glutaraldehyde solution (configured with PBS) overnight at 4 °C, followed by dehydration in a graded ethanol series (30%, 50%, 70%, 80%, 90% and 100%) for 15 min each. The 100% ethanol step was repeated twice before preparation for SEM.

### 2.7. Whole-Genome Sequencing

Following 20 days of selection, when resistance to gallium was established, isolation and sequencing of bacterial genomic DNA were performed at SeqCoast Genomics as previously described by Ewunkem et al. [28]. DNA was extracted from each population using the DNeasy 96 PowerSoil Pro QIAcube HT Kit according to the manufacturer’s instructions. Genomic libraries were produced using the Illumina DNA Prep tagmentation kit and IDT For Illumina Unique Dual Indexes according to the manufacturer ’s manuals. Read demultiplexing, read trimming and run analytics were performed using DRAGEN v4.2.7, an on-board analysis software system on the NextSeq2000.

### 2.8. Statistical Analysis

Statistical analysis of 24 h growth in response to increasing concentrations of gallium, iron, silver, tetracycline and chloramphenicol was performed using the General Linear Model, utilizing SPSS version 23 (SPSS Inc., Armonk, NY, USA). All graphs in this paper were made via Graphpad version 8.1.

## 3. Results

### 3.1. S. aureus Resistance in Gallium (III) Nitrate

**Figure 2.**
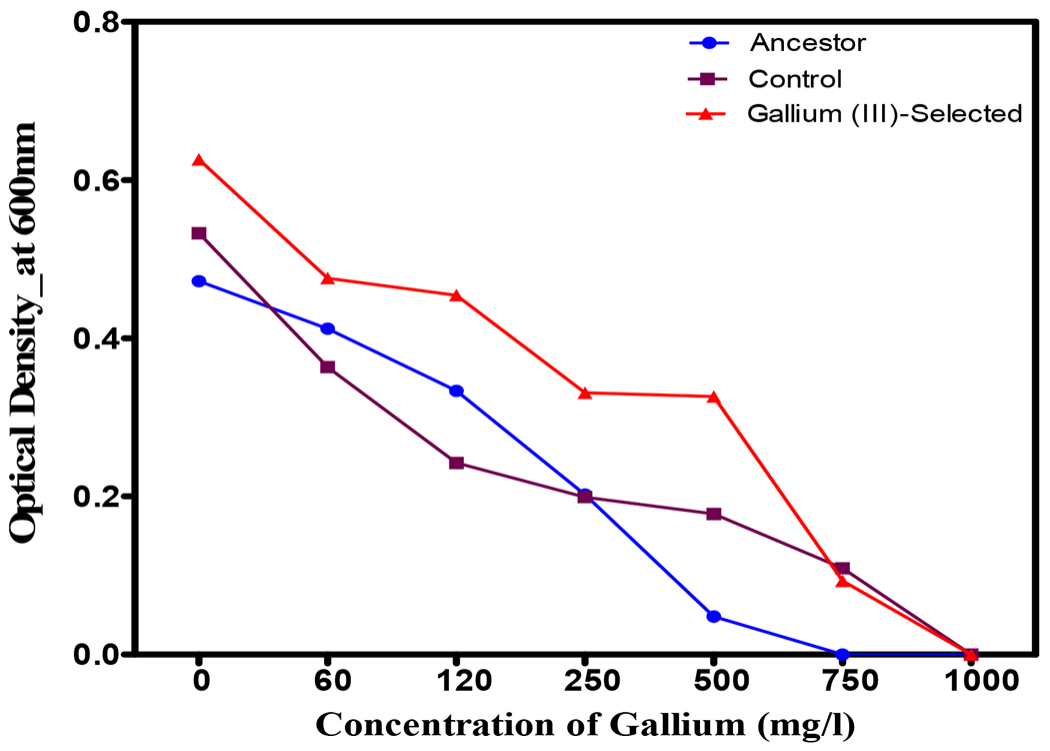
The mean of 24 h growth for *S. aureus* populations in increasing concentrations of gallium (III) after 30 days of evolution. Gallium (III)-selected bacteria showed significant growth compared to the control, followed by the ancestors. Gallium (III)-selected populations of *S. aureus* were exposed to 300 mg/L of gallium for 30 days in nutrient agar broth. Controls populations of *S. aureus* were grown in nutrient broth without gallium. Ancestral populations of *S. aureus* were grown in nutrient broth for 24 h.

After 20 days of selection, all populations showed a reduction in growth with increasing concentrations of gallium (III) nitrate. However, the gallium (III)-selected population displayed greater growth across all concentrations of gallium (III) nitrate (Figure 2) compared to the control and ancestral populations. The gallium (III)-resistant population showed significant (*p* < 0.0001) growth across concentrations from 0 to 750 mg/L, with greater optical densities, compared to the ancestral and the control populations.

### 3.2. Phenotypic Changes in the Presence of Heavy Metals

**Figure 3.**
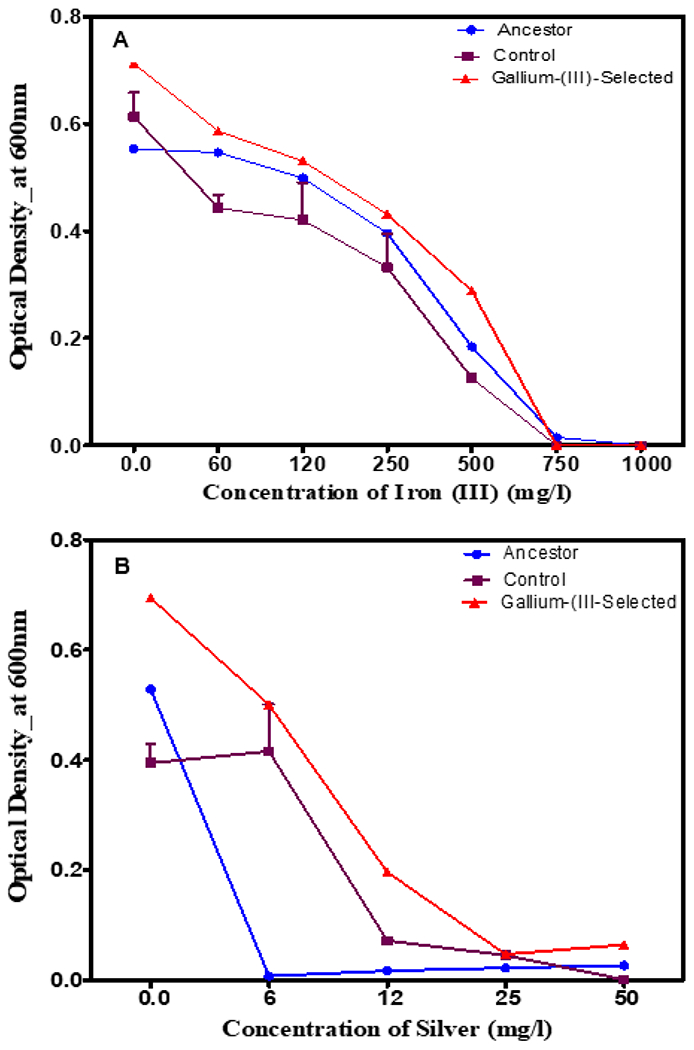
(**A**) The mean and SE of 24 h growth for populations in increasing concentrations of iron (III) up to 1000 mg/L after 20 days of evolution. Gallium (III)-selected bacteria showed significant growth compared to the control and the ancestor (**B**) The mean and SE of 24 h growth for populations in increasing concentrations of silver up to 50 mg/L after 20 days of evolution. The gallium (III)-selected bacteria showed significant growth compared to the controls, followed by the ancestors.

The gallium (III)-selected, control and ancestral populations were assessed for general metal resistance to determine potential pleiotropic effects associated with gallium (III) resistance across increasing concentrations of 0–750 mg/L of iron (III) nitrate and 0–50 mg/L of silver nitrate (Figure 3A,B). The gallium (III)-selected populations showed significantly (*p* < 0.0001) greater growth compared to the controls and the ancestors in iron (III) (Figure 3A) and silver (Figure 3B) across concentrations from 0 to 750 mg/L and from 0 to 50 mg/L, respectively. All populations showed a reduction in growth with increasing concentrations of metals and therefore demonstrated a significant concentration interaction between iron (III), gallium and silver.

### 3.3. Phenotypic Changes in the Presence of Traditional Antibiotics

**Figure 4.**
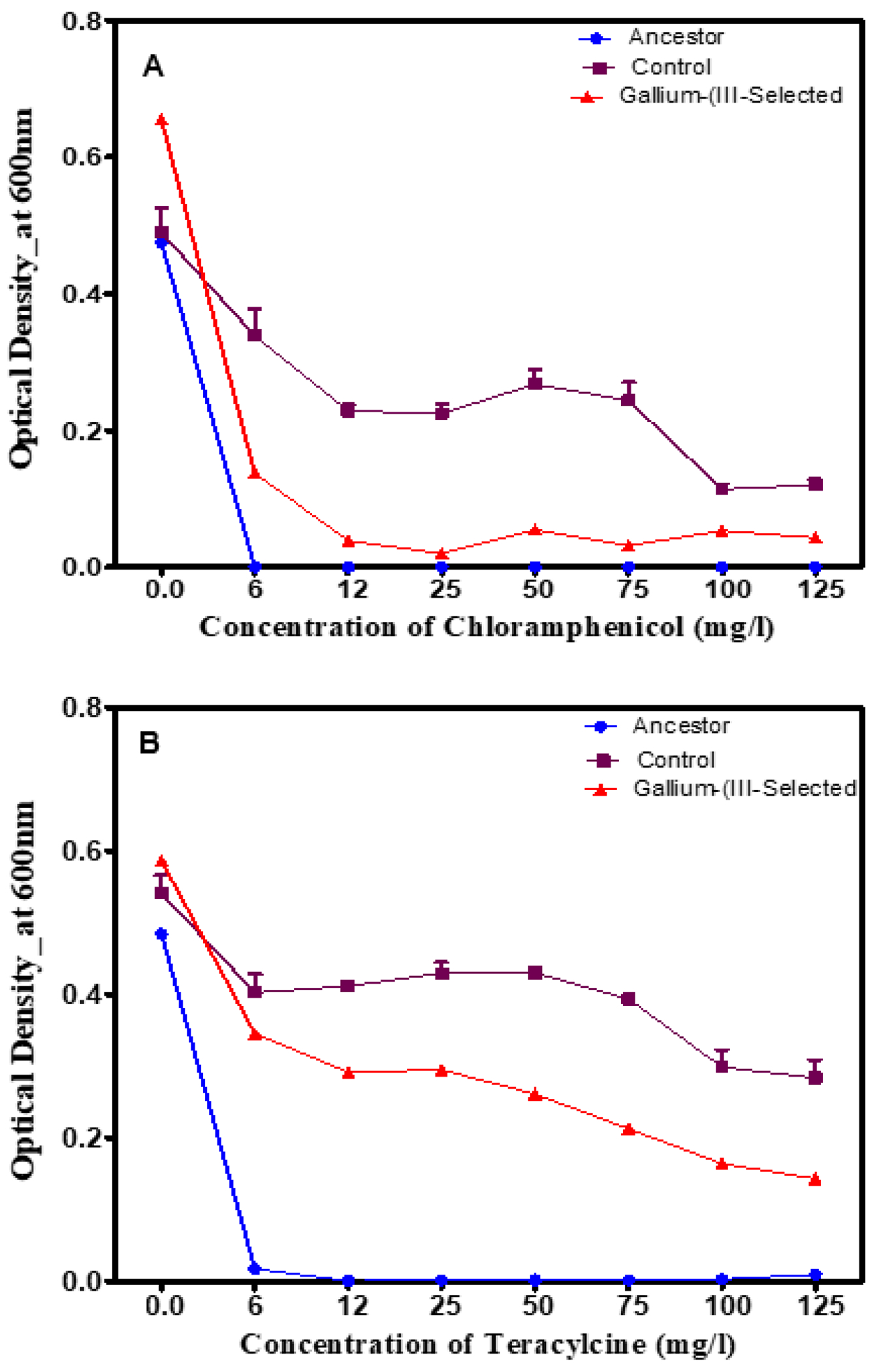
(**A**) The mean and SE of 24 h growth for populations in increasing concentrations of chloramphenicol up to 125 mg/L after 20 days of evolution. Gallium (III)-selected bacteria showed significantly inferior growth compared to the controls and the ancestors. (**B**) The mean and SE of 24 h growth for populations in increasing concentrations of tetracycline up to 125 mg/L after 20 days of evolution. The growth of the gallium (III)-selected bacteria was significantly inferior compared to the controls, followed by the ancestors.

Typically, metal resistance is known to co-select for antibiotic resistance; therefore, this study assessed the growth of gallium (III)-resistant bacteria following in two traditional antibiotics (tetracycline and chloramphenicol). Figure 4 shows the 24 h growth across increasing concentrations (0–125 mg/L) of chloramphenicol (which inhibits protein synthesis) and tetracycline (which also inhibits protein synthesis) for the gallium (III)-selected versus the control and ancestral populations after 20 days of selection. The gallium (III)-selected populations showed inferior growth, which was highly statistically significant (*p* < 0.0001) across concentrations from 0 to 250 mg/L. The growth of all the tested populations decreased significantly (*p* < 0.001) in increasing concentrations of tetracycline and chloramphenicol (Figure 4).

### 3.4. Phenotypic Changes in the Presence of Polyfluorinated Compounds

**Figure 5.**
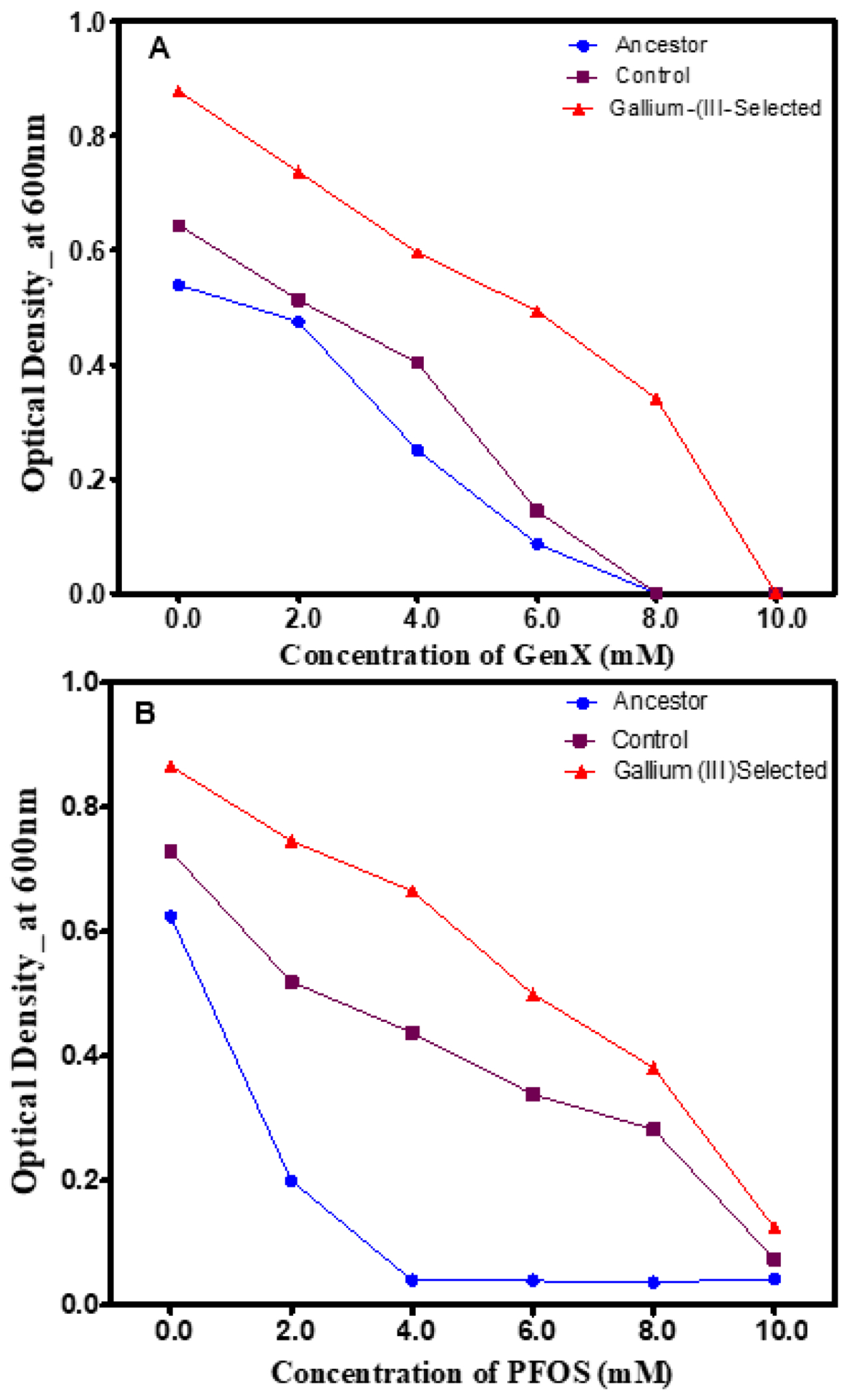
(**A**) The mean and SE of 24 h growth for populations in increasing concentrations of GenX up to 10 nM after 20 days of evolution. Gallium (III)-selected bacteria showed significantly higher growth rates compared to the control, followed by the ancestor (**B**) The mean and SE of 24 h growth for populations in increasing concentrations of PFOS up to 10 nM after 20 days of evolution. The gallium (III)-selected bacteria showed significantly higher growth rates compared to the control, followed by the ancestor.

Given the challenge of biological degradation of per- and polyfluoroalkyl substances, this study evaluated the growth of gallium (III)-selected bacterial, control and ancestral populations in the presence of increasing concentrations of two polyfluoroalkyl substances (or “forever chemicals”). Figure 5 shows 24 h growth across increasing concentrations (0–1000 mg/L) of GenX, a synthetic chemical compound, and perfluorooctanesulfonic acid (PFOS) after 20 days of selection. The gallium (III)-selected populations displayed superior growth, which was highly statistically significant (*p* < 0.0001) across concentrations from 0 to 8 Mm of GenX and PFOS compared to the control and ancestral populations (Figure 5A,B).

### 3.5. Scanning Electron Microscopy (SEM)

**Figure 6.**
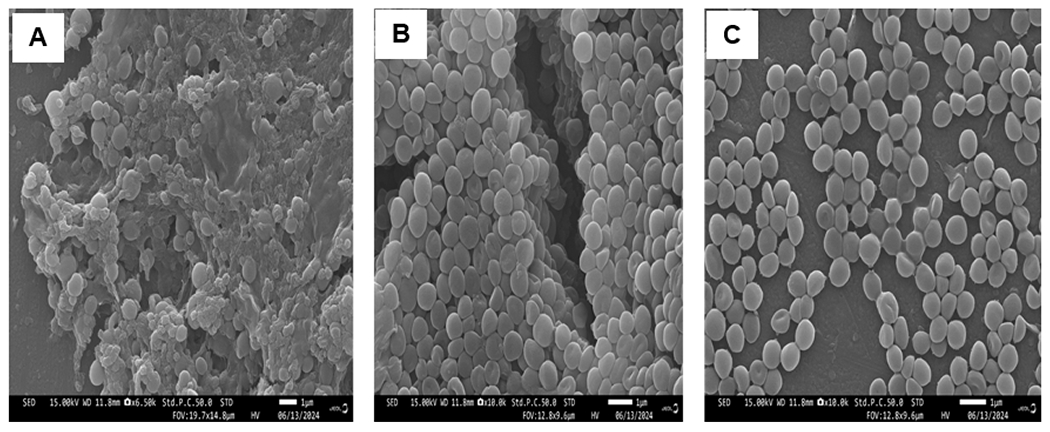
Scanning electron microscopy investigations of gallium (III)-selected *S. aureus*. Ancestral (**A**) and control (**B**) bacterial populations were intact, smooth and spherical, while 20 days of selection in gallium (III) nitrate caused the bacterial populations to appear oval, triangular, rod-like, as club-shaped rods and vibrio-shaped (**C**).

The electron micrographs obtained by SEM of the gallium (III)-selected bacterial, control and ancestral populations are shown in Figure 6. The micrographs showed that the surfaces of the control and ancestral *S. aureus* cells displayed characteristics typical of the surfaces of native cells, such as smooth, intact and round cocci, while the gallium (III)-selected bacteria treated with gallium (III) underwent a wide variety of cell morphological changes in response to gallium. Interestingly, some gallium-selected cells appeared oval, triangular, rod-like, as club-shaped rods and vibrio-shaped.

### 3.6. Genomic Results

To determine the effect of gallium selection on *S. aureus* genomic variations, we sequenced replicates from each selected population and defined a major polymorphism as F > 0.4, while a minor polymorphism was defined as a variant at a significantly lower frequency (F < 0.4). Table 1 shows polymorphisms in the gallium (III)-resistant populations (G_1_–G_5_) at day 20. The gallium (III)-resistant populations displayed significant polymorphisms in *sfaA/sfaD, KQ76_RS01520*, fmtA and *KQ76_RS08360*. Of the significant polymorphisms, all populations showed mutations in *sfaA/sfaD, KQ76_RS01520, fmtA, KQ76_RS08360, dltB* and *hssR*. Minor polymorphisms (F < 0.4) were observed in *dltB, KQ76_RS13825, KQ76_RS13475, purS, KQ76_RS12180, graR, KQ76_RS07375, KQ76_RS09255, KQ76_RS11185, vraE, KQ76_RS02795, capA, KQ76_RS04220, KQ76_RS05175/KQ76_RS05180, KQ76_RS01815* and *thrS*. Descriptions of these genes are given in Table 2.

The genomic variants found in the control populations (C1–C5) are listed in Table 3. Major polymorphisms in *pstC* and *KQ76_RS04365* were observed in all the replications (C1–C5). There were minor polymorphisms in *KQ76_RS13020*, PP2C family protein *KQ76_RS10525, ald/KQ76_RS08720, KQ76_RS12955, pxpB/greA, KQ76_RS06325, icaR, KQ76_RS10985, mprF, rsp, mutS, KQ76_RS11280/KQ76_RS11285 M23, KQ76_RS13825, KQ76_RS12905, KQ76_RS04770, KQ76_RS13475, smpB, mnmG, KQ76_RS12190/rsp, KQ76_RS11175, gltB* and *KQ76_RS01360/lip2*. Descriptions of these genes are given in Table 2. The control and gallium (III)-selected populations shared the following polymerphisms: *hssR, KQ76_RS13020, mnmG, KQ76_RS04770, KQ76_RS12955, KQ76_RS01360/lip2, KQ76_RS10985, gltB, rsp, ylqF, KQ76_RS11280/KQ76_RS11285, smpB* and *KQ76_RS11175*.

## 4. Discussion

Bacterial resistance to antibiotics is a serious threat to human health leading to more severe illnesses and increased mortality rates; this is considered an urgent public health threat by the World Health Organization (WHO) [31]. As rates of antibiotic-resistant bacterial infections soar, scientists are increasingly looking at the potential of metals as alternatives to combat these bacterial infections. Gallium is gaining attention because it targets alternative cellular processes, such as bacterial nutrition and metabolism [32]. Gallium has demonstrated bactericidal activity against *Streptomyces pilosus, Pseudomonas aeruginosa, Klebsiella pneumoniae, Escherichia coli* and *Staphylococcus aureus* [33–35]. Gallium exerts its bactericidal activity by interfering with enzymatic processes dependent on iron, thus making it hard for bacteria to evolve resistance to gallium [9,36–39]. A reduction in gallium uptake would reduce iron availability, which is critical for bacterial growth [40]. The fact that gallium mimics iron and interferes with multiple functions led us to hypothesize that *Staphylococcus aureus* may evolve resistance to gallium at high concentrations, as with successful antibiotics. In this study, we found that *S. aureus* evolved resistance to excess gallium (III) after 20 days, suggesting the presence of genetic adaptations, for example, efflux pumps, sequestration and enzymatic detoxification, essentially enabling them to expel or neutralize the harmful metals within their cells [41]. Previously, gallium (III) resistance acquired via transposon inactivation and mutations associated with iron metabolism has been investigated in Gram-negative bacteria [20,32]. Resistance to gallium (III) may not correlate with resistance to other metals, since the resistance pathway is largely specific to iron metabolism [42]. Other metals have different modes of action; for example, silver binds to membrane proteins, disrupting cell structure [43].

Pleiotropy is well known in the case of metals and antibiotics. The co-selection of metal and antibiotic resistance results from similarities between mechanisms, which include a reduction in cell wall permeability, substance alteration and efflux [28,44,45]. Here, gallium (III) selection showed a correlation with resistance to excess iron (III) nitrate and silver nitrate, but not tetracycline or chloramphenicol. The correlated response to iron (III) is not surprising because gallium (III) and iron (III) are chemically similar due to their similar ironic radii and charges, which enables gallium to replace iron in certain interactions [46,47]. Furthermore, all gallium (III)-resistant populations showed a mutation in staphyloferrinA export MFS transporter/D ornithine citrate ligase (*sfaA/sfaD*). *sfaA/sfaD* is known to actively pump staphyloferrin, a type of siderophore used to acquire iron from the environment, out of the bacterial cell. This process essentially exports the iron-chelating molecule to facilitate iron uptake when iron levels are low in the surrounding environment and is crucial for bacterial survival in iron-limited conditions [48]. By exporting staphyloferrin, gallium (III)-resistant *S. aureus* can efficiently acquire iron, which is essential for various cellular processes, including growth and replication [49]. A similar phenomenon was also reported in gallium (III)-resistant *E. coli* [15]. In this study, genomic analysis identified a hard selective sweep in the ferric citrate outer membrane transporter (*fecA*), known to mediate the transport of iron across the outer membrane, in the same manner as other siderophore transporters [50]. These results confirmed that gallium regulates iron membrane transport because it closely mimics iron in terms of its ionic radius, allowing it to be taken up by the same cellular mechanisms that transport iron. This phenomenon is often used in research and potential therapeutic applications to disrupt iron metabolism in cancer cells and bacteria. The mechanism of gallium (III) resistance to silver is not fully elucidated because selection in gallium (III) confers resistance to silver in the absence of selection of the genes *cusS* and *ompR*, which play a major role in conferring resistance [51,52]. In addition, resistance to silver may also occur through efflux of silver in cells [53]. Interestingly, in this study, genomic analysis identified polymorphisms in the magnesium transporter CorA family protein (*KQ76_RS12180*), NADP-dependent oxidoreductase (*KQ76_RS11185*) and the peptide resistance ABC transporter permease subunit (*vraE*), which play key roles in many metabolic pathways, including transport, efflux and detoxification [54–56]. In contrast, the gallium (III)-selected populations showed inferior growth to the control populations in tetracycline and chloramphenicol. The sensitivity of gallium (III)-resistant strains to tetracycline and chloramphenicol could be due to the absence of antibiotic-resistance genes. Studies have shown that bacteria which develop resistance to metals are also more likely to acquire resistance to antibiotics due to the often-shared genetic mechanisms [57]. Contrarily, mutations in genes associated with antibiotics, as in the case of acyltransferase family protein (*KQ76_RS04365*), were seen in all the replicates (C1–C5) of the control population. Pearson et al. [58] asserted that acyltransferase family protein (*KQ76_RS04365*) is essential for the biosynthesis of antibiotics and critical for regulating membrane biogenesis [58]. Additionally, the phenotypical changes of the control populations in tetracycline and chloramphenicol can also be attributed to genes associated with stress responses, cell division, detoxification and cell growth. Three genes—the phosphate ABC transporter permease subunit (*pstC*), PP2C family protein serine/threonine phosphatase (*KQ76_RS10525*) and 5 oxoprolinase subunit PxpB/transcription elongation factor (*pxpB/greA*)—showed major polymorphisms in the control population. The phosphate ABC transporter imports nutrients and exports toxic substances [59]. PP2C family protein serine/threonine phosphatase (*KQ76_RS10525*) controls many biological processes, including stress responses, development, cell division, virulence, and cell growth and death [60]. The 5 oxoprolinase subunit PxpB/transcription elongation factor (*pxpB/greA*) plays a crucial role in the metabolism of 5-oxoproline in bacterial cells and potentially affects their growth and survival under certain conditions [61].

Bacteria are ubiquitous and can grow in a variety of conditions, including air, water and soil. Some chemical manufacturing facilities and factories contaminate the environment by introducing polyfluoroalkyl compounds (also known as “forever compounds”) into air, water and soil. Due to their extremely slow breakdown rate, polyfluoroalkyl compounds persist in the environment, causing significant health issues in humans and animals, even at low levels of exposure [22]. Therefore, finding ways to degrade polyfluoroalkyl compounds is critical to mitigate environmental contamination and protect public health. Recent findings have shown that certain bacterial species, like *Desulfovibrio aminophilus* and *Sporomusa sphaeroides*, can break down polyfluoroalkyl compounds by cleaving key chemical bonds within the molecules, essentially allowing for the degradation of these persistent pollutants [62].

When bacteria grow in toxic waste, they can break down the waste through a process called bioremediation [63]. Gallium (III)-selected bacteria may help remove notorious polyfluoroalkyl compound chemicals. In this study, 24 h growth assays showed that the gallium (III)-selected populations displayed greater growth compared to the controls and the ancestors (gallium (III)-selected > controls > ancestor) in the polyfluoroalkyl compounds (GenX and PFBS) tested, boosting hopes that gallium-resistant bacteria might someday help remove these notoriously pervasive pollutants from the environment. The survival of these populations in GenX and PFBS could have been due to their ability to degrade Genx and PFBS by cleaving key chemical bonds within the molecules, essentially rendering them harmless. Additionally, the populations could have survived in polyfluoroalkyl compounds by evolving mechanisms to either actively pump out the toxins or modify their cell membranes to reduce permeability to the chemicals. These adaptations often involve genetic changes that allow them to sense and respond to the presence of toxic substances. For example, mutations in the response regulator transcription factor GraR/ApsR (*graR*) in gallium (III)-selected populations activated genes needed to survive in harsh environments [64]. Mutation in general stress protein (*KQ76_RS01815*) helps bacteria survive under a wide range of stressful conditions and cope with adverse conditions by activating a coordinated response, known as the “general stress response (GSR)”, which allows the bacteria to adapt to changing environments [65]. We also identified three significant polymorphisms associated with stress and detoxifications: response regulator transcription factor (*GraR/ApsR*), which activates genes needed to survive in harsh environments like high-temperature or low-pH conditions [64]; hypothetical protein (*KQ76_RS09255*), which plays a role in how bacteria adapt to their hosts [66]; and NADP dependent oxidoreductase (*KQ76_RS11185*), which plays a key role in many metabolic pathways and has a variety of applications, including biodegradation, detoxification and chemical synthesis [56]. It is important to note that the control populations also grew in Genx and PFBS, suggesting that they developed mechanisms to tolerate and even accumulate these toxic substances on their thick cell walls, allowing them to survive in such harsh conditions [67]. One notable observation was the presence of mutations in genes associated with efflux, detoxification and cell wall biosynthesis in these populations. This point is further elaborated on and explained in the paragraphs below.

Mutation in bacteria can alter the amino acid sequence of a protein involved in cell division, leading to changes in morphology [68]. In this study, for the first time, we examined the interaction of gallium (III) nitrate with *S. aureus* cells by SEM. Twenty days of selection in gallium (III) nitrate, to our surprise, resulted in variation in cell shapes, while the control and ancestral cells appeared perfectly spherical. It can be argued that long-term exposure to unfavorable conditions induces stress responses that impact cell size and shape for adaptation to the challenging environments [69]. Furthermore, stressors can perturb bacterial morphology by altering cell wall teichoic acid, binding to cross-linked peptidoglycans to interfere with cell wall maturation [70,71]. Another major factor that influences morphology is mutations in genes. Mutations can directly alter the morphology of a bacterium, suggesting that shape is important enough to merit regulation [72]. Several genomic variants involved in conferring gallium resistance were genes associated with cell walls. For example, mutation in PG:teichoic acid D alanyltransferase (*dltB*) (a key component of the Gram-positive bacterial cell wall) significantly impacts the cell wall’s charge and stability, influencing factors like bacterial virulence and immune response [73].

Interestingly, gallium (III) resistance seems to play a vital role in adhesion, virulence and nucleic acid synthesis, as gallium-resistant populations displayed mutations in teichoic acid D Ala esterase (*fmtA*), adenine phosphoribosyltransferase (*KQ76_RS08360*), ECF-type riboflavin transporter substrate-binding protein (*KQ76_RS13825*), glutathione peroxidase (*KQ76_RS13475*) and the phosphoribosylformylglycinamidine synthase subunit (*purS*). Mutation in teichoic acid D Ala esterase (*fmtA*) contributes to virulence by facilitating adhesion to host cells and evading immune defenses [74]. It is critical to understand the role of virulence associated with gallium (III) in vivo because it demonstrates how gallium (III) interacts with a living host, thus providing a more accurate understanding of its disease-causing capabilities. The virulence potential of gallium (III) can be tested in vivo by infecting an animal model, for example, mice, and then monitoring tissue damage and the mortality rate. Adenine phosphoribosyltransferase (*KQ76_RS08360*) plays a role in the recycling of nucleotides in bacteria [75]. ECF-type riboflavin transporter substrate-binding protein (*KQ76_RS13825*) allows the transport of essential vitamins from the surrounding medium for growth and metabolism [76,77]. Glutathione peroxidase (*KQ76_RS13475*) protects cells from oxidative damage and plays a role in bacterial virulence and pathogenicity [78]. The phosphoribosylformylglycinamidine synthase subunit (*purS*) plays a key role in the de novo purine synthesis pathway [79]. DUF3169 family protein (*KQ76_RS01520*) is essential for the secretion and transport of proteins [80].

The control populations displayed a total of 27 putative polymorphisms, of which 13 were shared with gallium (III)-selected populations. All the control populations carried major polymorphism (F > 0.4) sweeps in acyltransferase family protein (*KQ76_RS04365*), PP2C family protein serine/threonine phosphatase (*KQ76_RS1052*) and 5 oxoprolinase subunit PxpB/transcription elongation factor (*pxpB/greA*). Acyltransferase family protein (*KQ76_RS04365*) and PP2C family protein serine/threonine phosphatase (*KQ76_RS1052*) play important roles in regulating membrane biogenesis stress responses, development, cell division, virulence, and cell growth and death [58,60,61]. Three polymorphisms were detected in four of the five control populations. Polymorphisms were detected in the phosphate ABC transporter permease subunit (*pstC)*, alpha/beta hydrolase (*KQ76_RS13020)* and cell elongation protein (*cozEb*). These genomic variants are involved in cell division, antimicrobial virulence, nutrients and export of toxic substance regulation [59,81,82]. Additionally, four mutations unique to the control populations were detected at lower frequencies (F < 0.4). These mutations included the ica operon transcriptional regulator (*icaR*), hypothetical protein (*KQ76_RS09255*), alanine dehydrogenase/universal stress protein (*ald/KQ76_RS08720*), ECF-type riboflavin transporter substrate-binding protein (*KQ76_RS13825*), bifunctional lysylphosphatidylglycerol flippase/synthetase (*mprF*), DNA mismatch repair protein (*mutS*), glutathione peroxidase (*KQ76_RS13475*) and the YbgA family protein/AraC family transcriptional regulator (*KQ76_RS12190/rsp*). Not surprisingly, these mutations are known to play roles in regulating biofilm formation, transport across membranes, replication oxidative damage and gene expression [76,78,83,84]. The control and gallium (III)-selected populations showed polymorphisms in the DNA-binding heme response regulator (*hssR*), alpha/beta hydrolase (*KQ76_RS13020*), the tRNA uridine 5 carboxymethylaminomethyl(34) synthesis enzyme (*mnmG*), ATP-binding protein (*KQ76_RS04770*), D lactate dehydrogenase (*KQ76_RS12955*), YjiH family protein/YSIRK domain-containing triacylglycerol lipase Lip2/Geh (*KQ76_RS01360/lip2*), BglG family transcription antiterminator (*KQ76_RS10985*), the glutamate synthase large subunit (*gltB*), the AraC family transcriptional regulator (*rsp*), ribosome biogenesis GTPase (*ylqF*), M23 family metallopeptidase/HAD IIB family hydrolase (*KQ76_RS11280/KQ76_RS11285*), SsrA-binding protein (*smpB*) and the BCCT family transporter (*KQ76_RS11175*). Polymorphisms in these genes are crucial for adaptation, antimicrobial activity, translation, import of nutrients, energy, colonization and infection, virulence, antimicrobial potential, and osmoregulation, which enable *S. aureus* to survive and replicate within their hosts [59,81,85–97]. This may explain the similarities in phenotypical changes between the control and treated populations in the presence of increasing concentrations of polyfluorinated compounds. Polymorphisms in both populations suggest the presence of genetic variations that exist naturally within *S aureus* [98]. Furthermore, the observed polymorphisms are not directly caused by gallium (III) and could be naturally occurring variations within *S. aureus* [99]. However, the genes were in different locations, potentially leading to different effects on the resulting proteins [100]. Additional experiments are needed to explore the potential functional implications of these polymorphisms. This study used *Staphylococcus aureus* (ATCC 25923); different strains of *S. aureus* could yield different results. Also, in our future studies, we intend to measure changes in gene expression and ultrastructure changes associated with gallium (III) resistance utilizing RNA sequencing and transmission electron microscopy (TEM), respectively.

## 5. Conclusions

With the rise in bacterial resistance to antibiotics, gallium is a promising treatment for multidrug-resistant pathogens. Gallium is a potential antimicrobial because it disrupts bacterial metabolism by mimicking iron and substituting itself into key iron-dependent enzymes [101]. In this study, we showed that gallium resistance in *S. aureus* occurred after 20 days of selection in gallium (III) nitrate due to specific mutational and morphological changes increasing the ability to grow in heavy metals and polyfluorinated compounds. All the replicates of gallium (III)-resistant strains showed polymorphisms in staphyloferrinA export MFS transporter/D ornithine citrate ligase (*sfaA/sfaD*), DUF3169 family protein (*KQ76_RS01520*) and teichoic acid D Ala esterase (*fmtA*), suggesting strong evidence of the crucial roles these genes play in conferring gallium resistance. Moreover, the genomic changes led to noticeable alterations in cell morphology. In contrast, the gallium-selected *S. aureus* displayed inferior growth in antibiotics compared to the control population. Our results highlight significant implications for human health, ecosystems and environmental remediation.

## Figures and Tables

**Table 1.** Selective sweeps in gallium (III)-resistant populations at day 20.

Gene	Position	Mutation	G1	G2	G3	G4	G5
*sfaA/sfaD*	2,228,365	Intergenic (−35/−67)	0.767	0.692	0.731	0.758	0.755
*KQ76_RS01520*	342,330	I180I* (ATT→ATC)	0.678	0.62	0.645	0.673	0.631
*fmtA*	1,017,325	Q304* (CAA→TAA)	0.583	0.653	0.599	0.624	0.539
*KQ76_RS08360*	1,700,478	G59S* (GGC→AGC)	0.367	0.656	0.641	0.541	0.538
*dltB*	860,322	E344K* (GAA→AAA)	0.325	0.313	0.37	0.266	0.363
*hssR*	2,389,192	R188P* (CGA→CCA)	0.327	0.32	0.382	0.328	0.339
*rsp*	2,412,575	G84D* (GGT→GAT)	0	0	0	0	0.330
*KQ76_RS11280/KQ76_RS11285*	2,252,747	Intergenic (−54/+157)	0	0.299	0	0.281	0.325
*KQ76_RS13020*	2,574,726	G69A* (GGC→GCC) ‡	0.274	0.322	0.355	0	0.318
*KQ76_RS13825*	2,746,925	A172G* (GCT→GGT)	0.352	0	0.36	0.246	0
*KQ76_RS12955*	2,564,194	A17P* (GCA→CCA)	0.298	0	0	0	0.281
*gltB*	453,752	Pseudogene (4457/4500 nt)	0	0	0	0	0.281
*KQ76_RS13475*	2,665,478	E162V* (GAA→GTA)	0	0	0	0	0.278
*purS*	1,027,271	A87P* (GCA→CCA)	0	0.27	0.239	0.262	0.272
*KQ76_RS11175*	2,233,551	T39S* (ACT→AGT)	0.27	0	0	0	0
*KQ76_RS10985*	2,190,680	T500S* (ACG→TCG)	0.255	0.275	0	0.237	0.262
*KQ76_RS12180*	2,408,435	L213I* (TTA→ATA)	0	0.254	0	0	0.262
*graR*	673,306	A185P* (GCA→CCA)	0.264	0	0	0	0.26
*KQ76_RS07375*	1,536,460	Y112* (TAT→TAA)	0	0.275	0	0	0
*KQ76_RS09255*	1,890,405	S137T* (AGT→ACT)	0.254	0.266	0.265	0	0
*KQ76_RS11185*	2,236,063	R52P* (CGT→CCT)	0.272	0	0	0	0.251
*KQ76_RS04770*	986,849	K56* (AAA→TAA)	0.281	0	0.254	0	0
*ylqF*	1,213,003	E184D* (GAG→GAC)	0.288	0.313	0.237	0.26	0
*smpB*	810,694	M1K* (ATG→AAG) †	0	0.29	0	0	0
*vraE*	2,766,496	I591I* (ATA→ATT)	0.268	0	0	0	0
*KQ76_RS02795*	596,886	A144V* (GCA→GTA)	0	0	0.235	0	0
*KQ76_RS01360/lip2*	311,944	Intergenic (−69/−348)	0	0	0	0.234	0
*capA*	116,233	V151M* (GTG→ATG)	0	0	0	0.233	0
*KQ76_RS04220*	871,433	T26I* (ACA→ATA)	0	0	0	0.225	0
*KQ76_RS05175/KQ76_RS05180*	1,066,987	Intergenic (−157/+27)	0.254	0	0	0	0
*KQ76_RS01815*	391,361	S36I* (AGT→ATT)	0	0.291	0	0	0
*mnmG*	2,776,116	H117Q* (CAT→CAA)	0	0.276	0	0	0
*thrS*	1,740,864	L149* (TTA→TAA)	0	0	0	0.224	0

Notes: After 20 days of selection, all populations were subjected to whole-genome resequencing with sequence alignments and variant calling using breseq 0.30.0. The genes/mutations and frequencies are reported. Gallium (III)-selected populations (G1–G5).

* Annotation gives meaning to a given sequence and makes it much easier to view and analyze its contents. Numbers in the last five columns represent the frequencies.

† Indicates a mutation or variant with a specific significance.

‡ Indicates a variant that has been flagged as potentially problematic or requiring further investigation. The underlined colors are the various mutations.

**Table 2.** Descriptions of genes.

*pstC*	Phosphate ABC transporter permease subunit
*KQ76_RS04365*	Acyltransferase family protein
*hssR*	DNA-binding heme response regulator
*KQ76_RS10525*	PP2C family protein–serine/threonine phosphatase
*pxpB/greA*	5-oxoprolinase subunit PxpB/transcription elongation factor
*KQ76_RS13020*	Alpha/beta hydrolase
*icaR*	Ica operon transcriptional regulator
*KQ76_RS09255*	Hypothetical protein
*ald/KQ76_RS08720*	Alanine dehydrogenase/universal stress protein
*mnmG*	tRNA uridine-5-carboxymethylaminomethyl(34) synthesis enzyme
*KQ76_RS04770*	ATP-binding protein
*cozEb*	Cell elongation protein
*KQ76_RS12955*	D-lactate dehydrogenase
*KQ76_RS01360/lip2*	YjiH family protein/YSIRK domain-containing triacylglycerol lipase Lip2/Geh
*KQ76_RS10985*	BglG family transcription antiterminator
*KQ76_RS13825*	ECF-type riboflavin transporter substrate-binding protein
*gltB*	Glutamate synthase large subunit
*rsp*	AraC family transcriptional regulator
*ylqF*	Ribosome biogenesis GTPase
*mprF*	Bifunctional lysylphosphatidylglycerol flippase/synthetase
*mutS*	DNA mismatch repair protein
*KQ76_RS11280/KQ76_RS11285*	M23 family metallopeptidase/HAD IIB family hydrolase
*KQ76_RS12905*	ATP-binding cassette domain-containing protein
*smpB*	SsrA-binding protein
*KQ76_RS13475*	Glutathione peroxidase
*KQ76_RS12190/rsp*	YbgA family protein/AraC family transcriptional regulator
*KQ76_RS11175*	BCCT family transporter
*sfaA/sfaD*	StaphyloferrinA export MFS transporter/D-ornithine–citrate ligase
*KQ76_RS01520*	DUF3169 family protein
*fmtA*	Teichoic acid D-Ala esterase
*KQ76_RS08360*	Adenine phosphoribosyltransferase
*dltB*	PG:teichoic acid D-alanyltransferase
*KQ76_RS13825*	ECF-type riboflavin transporter substrate-binding protein
*KQ76_RS13475*	Glutathione peroxidase
*purS*	Phosphoribosylformylglycinamidine synthase subunit
*KQ76_RS12180*	Magnesium transporter CorA family protein
*graR*	Response regulator transcription factor GraR/ApsR
*KQ76_RS07375*	Phage major capsid protein
*KQ76_RS09255*	Hypothetical protein
*KQ76_RS11185*	NADP-dependent oxidoreductase
*vraE*	Peptide-resistance ABC transporter permease subunit
*KQ76_RS02795*	Uracil–DNA glycosylase
*capA*	Capsular polysaccharide-type 5/8 biosynthesis protein
*KQ76_RS04220*	FAD/NAD(P)-binding protein
*KQ76_RS05175/KQ76_RS05180*	Nramp family divalent metal transporter/YktB family protein
*KQ76_RS01815*	General stress protein
*thrS*	Threonine–tRNA ligase

Note: Color coding: Polymorphisms in the control populations are shown in grey; polymorphisms in the gallium (III)-selected populations only are shown in brown; polymorphisms in both the control and gallium (III)-selected populations are shown in green.

**Table 3.** Selective sweeps in control populations at day 20.

Gene	Position	Mutation	C1	C2	C3	C4	C5
*pstC*	1,384,254	S178C* (AGT→TGT)	0	0.728	0.685	0.73	0.709
*KQ76_RS04365*	907,688	N549I* (AAT→ATT)	0.625	0.66	0.61	0.68	0.653
*KQ76_RS10525*	2,102,321	G175E* (GGA→GAA)	0.417	0.463	0.399	0.378	0.39
*KQ76_RS13020*	2,574,726	G69A* (GGC→GCC)	0	0.314	0.31	0.367	0.468
*ylqF*	1,213,003	E184D* (GAG→GAC)	0	0.279	0	0.336	0
*KQ76_RS13020*	2,574,727	G69R* (GGC→CGC)	0.338	0	0.36	0.316	0
*hssR*	2,389,188	E187Q* (GAA→CAA)	0.349	0.30	0.41	0.316	0
*pxpB/greA*	1,672,825	Intergenic (−76/+250)	0.31	0.293	0.39	0.313	0.272
*icaR*	2,727,937	Q79* (CAA→TAA)	0.278	0	0.29	0	0
*KQ76_RS10525*	2,102,321	G175E* (GGA→GAA)	0.302	0	0	0	0
*KQ76_RS06325*	1,302,347	D54Y* (GAC→TAC)	0.293	0	0	0.296	0.259
*cozEb*	1,352,269	I247N* (ATT→AAT)	0	0.279	0.259	0.293	0.259
*mprF*	1,354,114	G299D* (GGT→GAT)	0	0	0	0.292	0.253
*pepF/yjbH*	941,253	Intergenic (+205/+255)	0	0	0	0.292	0
*KQ76_RS13825*	2,746,925	A172G* (GCT→GGT)	0	0.272	0	0.290	0.248
*KQ76_RS12190/rsp*	2,412,155	Intergenic (+1318/170)	0.246	0	0	0	0
*KQ76_RS10985*	2,190,680	T500S* (ACG→TCG)	0	0.279	0	0.290	0.256
*KQ76_RS09255*	1,890,405	S137T* (AGT→ACT)	0	0.245	0.286	0	0
*KQ76_RS12955*	2,564,194	A17P* (GCA→CCA)	0	0.251	0	0.287	0.31
*ald/KQ76_RS08720*	1,776,393	Intergenic (−57/−84)	0.349	0	0.286	0	0.341
*KQ76_RS04275/KQ76_RS04280*	881,138	Intergenic (+150/−158)	0.236	0	0	0.270	0.249
*mnmG*	2,776,116	H117Q* (CAT→CAA)	0	0.246	0.276	0	0
*ald/KQ76_RS08720*	1,776,393	Intergenic (−57/−84)	0	0	0.286	0.265	0
*KQ76_RS12955*	2,564,193	M16I* (ATG→ATC)	0	0	0.25	0	0
*KQ76_RS13475*	2,665,478	E162V* (GAA→GTA)	0.276	0	0	0	0
*mutS*	1,275,400	K479N* (AAG→AAC)	0	0	0	0	0.25
*rsp*	2,412,863	C180Y* (TGT→TAT)	0.246	0	0	0	0.251
*smpB*	810,694	M1K* (ATG→AAG)	0.264	0	0	0	0
*KQ76_RS01360/lip2*	311,944	Intergenic (−69/−348)	0	0	0.247	0	0
*KQ76_RS10985*	2,190,680	T500S* (ACG→TCG)	0	0	0.247	0	0
*KQ76_RS04770*	986,858	Q59K* (CAA→AAA)	0	0.245	0	0	0.244
*KQ76_RS13825*	2,746,925	A172G* (GCT→GGT)	0	0	0.245	0	0
*gltB*	453,752	Pseudogene (4457/4500 nt)	0	0.238	0.243	0	0
*KQ76_RS12905*	2,554,550	V173L* (GTC→CTC)	0	0	0	0	0.247
*KQ76_RS11175*	2,233,551	T39S* (ACT→AGT)	0.23	0	0	0	0

Notes: After 20 days of selection, all populations were subjected to whole-genome resequencing with sequence alignments and variant calling using breseq 0.30.0. The genes/mutations and frequencies are reported. Control populations (C1–C5).

* Annotation gives meaning to a given sequence and makes it much easier to view and analyze its contents. Numbers in the last five columns represent the frequencies. The underlined colors are the various mutations.

## Data Availability

Data are contained within the article.
